# Molecular Dynamic Investigation of the Anisotropic Response of Aluminum Surface by Ions Beam Sputtering

**DOI:** 10.3390/mi12070848

**Published:** 2021-07-20

**Authors:** Chunyang Du, Yifan Dai, Chaoliang Guan, Hao Hu

**Affiliations:** 1 College of Intelligence Science, National University of Defense Technology, Changsha 410073, China; nature_cydu@vip.sina.com (C.D.); dyf@nudt.edu.cn (Y.D.); gchl_nudt@163.com (C.G.); 2 Laboratory of Science and Technology on Integrated Logistics Support, National University of Defense Technology, Changsha 410073, China; 3 Hu’nan Key Laboratory of Ultra-Precision Machining Technology, Changsha 410073, China

**Keywords:** aluminum, ion beam sputtering, morphology evolution, subsurface damage, molecular dynamics

## Abstract

Aluminum optics are widely used in modern optical systems because of their high specific stiffness and high reflectance. With the applied optical frequency band moving to visible, traditional processing technology cannot meet the processing precision. Ion beam sputtering (IBS) provides a highly deterministic technology for high-precision aluminum optics fabrication. However, the surface quality is deteriorated after IBS. The interaction between the bombard atoms and the surface morphology evolution mechanism are not clear, and systematic research is needed. Thus, in this paper, the IBS process for single crystal aluminum with different crystallographic orientations are studied by the molecular dynamics method. The ion beam sputter process is firstly demonstrated. Then, the variation of sputter yield of the three crystal faces is analyzed. The sputter yield difference of different crystal surfaces causes the appearance of the relief structure. Then, the gravel structure generates on the single crystal surfaces and dominates the morphology evolution. The state of the atom diffusion of the specific crystal surfaces will determine the form of the gravel structure. Furthermore, the form and distribution of subsurface damage and stress distribution of three different crystal surfaces are analyzed. Although there are great differences in defect distribution, no stress concentration was found in three workpieces, which verifies that the ion beam sputter is a stress-free machining method. The process of IBS and the mechanism of morphology evolution of aluminum are revealed. The regularity and mechanism will provide a guidance for the application of IBS in aluminum optics manufacture fields.

## 1. Introduction

With fine mechanical properties, light weight and high reflectivity, aluminum is widely used in optical systems in recent years, especially in the micro-satellites with extreme requirements for weight and volume [1,2,3]. Currently, the applied optical frequency band of aluminum optics is moving from infrared (IR) to visible (VIS), which brings a great challenge to fabrication [4]. For application in visible band, the aluminum optics should possess nanometer scale surface profile precision and subnanometer scale surface roughness. Usually, single-point diamond turning (SPDT) and magnetorheological finishing (MRF) are widely used in the fabrication of aluminum optics [5,6]. However, the precision of these methods cannot meet the requirements for visible light usage. Moreover, due to the properties of high chemical reactivity and low surface hardness, contact machining will cause the contamination of the aluminum surface, which is often associated with a reduction in the surface quality [7].

As a high-determined machining method, ion beam sputtering (IBS) achieves surface profile correction by removing materials through physic sputtering effects [8,9]. When receiving enough energy from bombarded atoms, the surface atoms will be sputtered from the surface. IBS is believed to possess the highest machining precision. Moreover, the whole process is conducted in a near-vacuum environment which will not cause contamination [10,11]. All the advantages show that IBS can be a better polishing method for visible range aluminum optics compared with contact polishing methods such as MRF. In recent years, more researches have been focused on the roughness and surface morphology revolution during IBS. Remarkable experimental work is conducted to demonstrate the formation of specific micro-topographies, such as ripples [12,13] and nanoparticles [14,15]. However, most researches are conducted on amorphous materials such as fused silica. There is a great lack of studies about aluminum. A systematic experiment on roughness evolution of different materials during IBS was carried out by C.M. Egert [16]. Aluminum shows poor performance on the decrease in roughness. The same phenomenon is also observed in the experiments conducted by our research group. Unlike traditionally used materials in IBS, peculiar relief and gravel structure emerge on the aluminum surface during IBS [17]. In order to realize the regulation of surface microscopic morphology and surface roughness, the process of IBS and mechanism of morphology evolution need to be revealed. However, experimentalists face the daunting task of characterizing the material removal and surface evolution of aluminum at nanoscale time and space. Thus, the mechanism is still unrevealed.

Molecular dynamics (MD) simulations play a key role in understanding the experimental results, revealing mechanism and predicting outcomes [18,19,20]. Remarkable works have been successfully conducted, revealing the mechanism of interaction between energy particles and many other materials. Using MD, Wang et al. reveal the elastoplastic transformation process of monocrystalline silicon material induced by ion implantation [21]. By optimizing the ion implantation, amorphous layer with microns’ scale can be generated. Xiao et al. have studied the surface damage evolution of nanoscale silicon caused by Ga-focused ion beam machining. Simulation results are in good agreement with the experimental results [22]. Xiao et al. have successfully revealed the material removal process and surface morphology evolution of single crystal silicon during IBS [23]. However, there is limited work related to the metal materials such as aluminum [24,25,26,27,28,29]. Moreover, the aluminum used in the IBS are usually in an alloy state. There are multiple crystal faces on the surface. In order to realize the regulation of surface microscopic morphology of aluminum, it is necessary to fully understand the mechanism in each crystallographic orientation through MD.

In this work, the IBS process and surface morphology evolution of three kinds of crystal surfaces are studied. In Section 2, the method of MD simulation, defect analysis, and visualization techniques are presented. In Section 3, the results of ion beam sputtering process and its mechanism, surface morphology evolution, sputtering yield, and subsurface damage are analyzed and discussed. Finally, a conclusion is summarized in Section 4. The results of this study will be beneficial to understanding the IBS of aluminum and promoting the application of IBS in the field of aluminum optics manufacture, which will significantly improve the machining efficiency and precision of aluminum optics.

## 2. Simulation Method

### 2.1. Simulation Description

**Figure 1:**
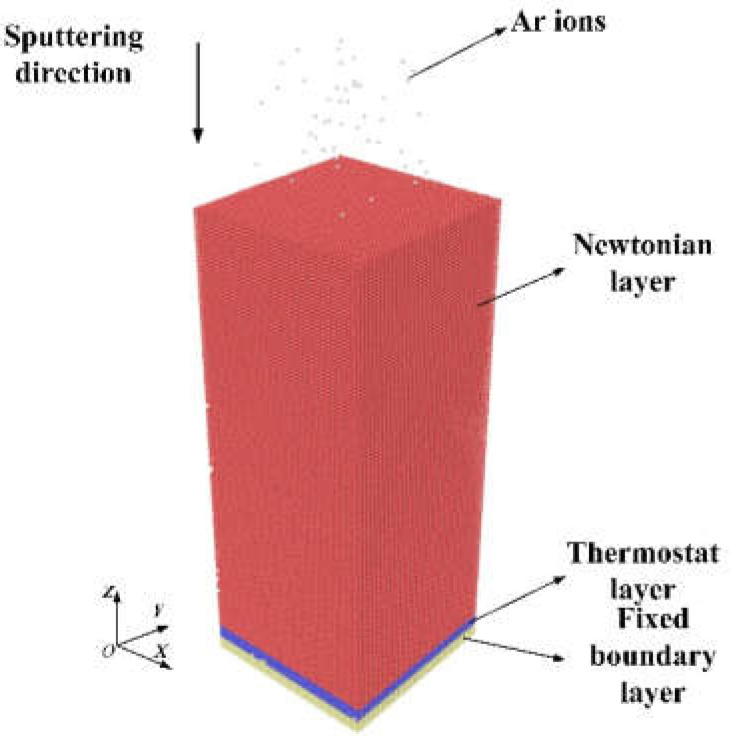
MD model of ion beam sputtering of single crystal aluminum.

The MD model of Ar ion sputtering consists of a single crystal aluminum workpiece and Ar atoms, as indicated in Figure 1. The aluminum workpiece has a dimension of 15, 15, and 38 nm in X, Y, and Z directions, respectively. To investigate the influence of crystal orientation, three aluminum workpieces with Al(110), Al(111), and Al(001) free surface in Z direction are considered. Ar atoms bombard the workpiece vertically in the Z direction. Because IBS is conducted in a near-vacuum environment, the influence of the environment can be ignored. As shown in Figure 1, the aluminum sample is divided into three regions: the region of boundary atoms to fix the sample in space, the region of thermostat atoms to imitate the heat dissipation, and the region of Newtonian atoms which obeys Newton’s second law [30,31,32,33]. The thickness of thermostat layer and boundary layer are both 1 nm. The initial temperature of the aluminum sample is maintained at 293 K. The periodic boundary condition is applied on the direction of X and Y to elimination of size effect.

During IBS, Ar is firstly ionized in the cavity and accelerated by screen-grid voltage. Then, a neutralizer is applied to the generated electron to neutralize the charge of Ar^+^. Thus, the Ar atoms will have the same bombardment speed as shown in Table 1. The Ar will bombard the workpiece surface in a steady stream. Thus, in this study, we adopt a continuous bombardment situation. First, *k* Ar atoms are distributed randomly in a 10 × 10 × 1 nm^3^ box to simulate specific ionic concentration in the cavity. Then, the 10 × 10 × 1 box will expand *n* times in the Z direction. For each 10 × 10 × 1 nm^3^ box, the Ar atoms in the box are randomly distributed. The total Ar atoms bombarding on the surface will be *k* × *n*. All the MD simulations are based on LAMMPS developed by Sandia National Laboratory (PO Box 5800, Albuquerque, NM, USA). The Ovito is utilized to perform visualization of MD simulation of the IBS process. The velocity-Verlet algorithm is applied to integrate Newton’s equations of motion with the time step of 1 fs. The common neighbor analysis (CNA) is used to identify the crystal structure during the ion sputtering. Firstly, the energy minimization is carried out by the conjugate gradient method to avoid the overlap of atomic position. Then, the temperature of workpiece is equilibrated to 293 K by the Nose–Hoover thermostat for 70 ps. Both the relaxation stage and sputtering simulation are performed in a microcanonical ensemble (NVE) [34,35,36]. The Ar atoms are placed at the height of 2 nm above the initial top surface of workpiece. Considering the commonly process parameters of IBS, the ion energy is chosen to be 500 eV and the incident angle is 90°. The total number of bombarded Ar atoms is 50. The ion dose is defined as the total number of Ar atoms trapped in workpiece divided by the upper surface area of workpiece. The simulated parameters are represented in Table 1.

### 2.2. Potential Description

The mixing potentials are used in the ion beam sputtering simulation. The interaction potentials between atoms are described as follow:

(1) For Ar–Ar atomic interaction, the Ziegler–Biersack–Littmark (ZBL) potential is adopted, which can be expressed as follow [37]:

(1) $$E_{{}^{{}_{ij}}}^{ZBL}=\frac{1}{4\pi \varepsilon_{0}}\cdot \frac{Z_{i}Z_{j}e^{2}}{r_{ij}}\phi (r_{ij}/a)+S(r_{ij}),$$

(2) $$a=\frac{0.46850}{Z_{i}^{0.23}+Z_{j}^{0.23}},$$

(3) $$\phi (x)=0.18175e^{-3.19980x}+0.50986e^{-0.94229x}+0.28022e^{-0.40290x}+0.02817e^{-0.20162x}.$$

where *e* is the electron charge, *ε*_0_ is the electrical permittivity of vacuum, and *Z_i_* and *Z_j_* are the nuclear charges of the two atoms. *S*(*r*) is the switching function.

(2) For Al–Al atomic interaction, the embedded-atom method (EAM) potential is adopted, which can be expressed as follow [38]:

(4) $$E_{i}=F_{a}(\sum_{j\neq i}\rho_{\beta }(r_{ij}))+\frac{1}{2}\sum_{j\neq i}\phi_{\alpha \beta }(r_{ij}).$$

where *E_i_* is the total energy, *F* is the embedding energy which is a function of the atomic electron density *ρ*, *ϕ* is a pair potential interaction, *α* and *β* are the element types of atoms *i* and *j*.

(3) For Al–Ar atomic interaction, the splicing potentials are adopted. ZBL, the second order polynomial function and Lennard–Jones (*LJ*) potentials are used for different atomic spacing, respectively, to construct the Ar–Al potential function. The *LJ* potential is expressed as follow:

(5) $$U_{ij}^{LJ}(r_{ij})=4\varepsilon [{\left(\frac{\sigma }{r_{ij}}\right)}^{12}-{\left(\frac{\sigma }{r_{ij}}\right)}^{6}]$$

where *ε* is the depth of the potential well, *σ* is the distance of zero potential. ZBL potential is used in the range of 0~0.31 nm and *LJ* potential in the range of 0.37~∞ nm. In the range of 0.31~0.37 nm, a second order polynomial function is used to join the two potentials [39].

## 3. Results and Discussion

### 3.1. Ion Beam Sputtering Mechanism

**Figure 2:**
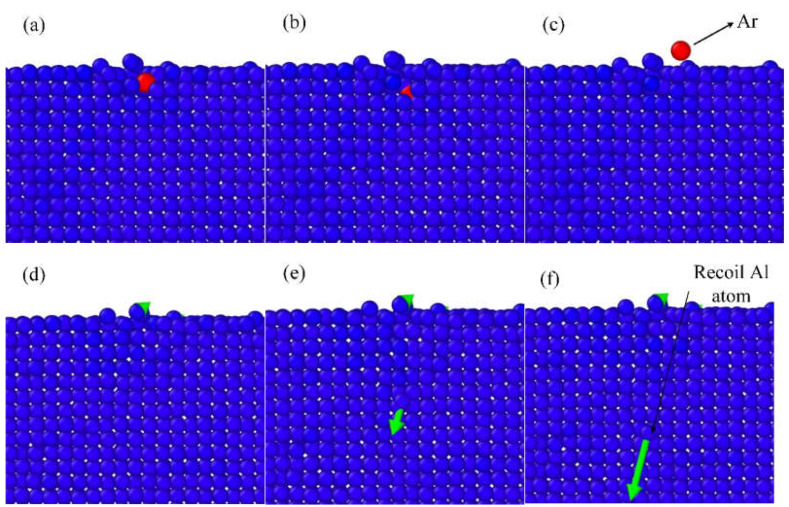
Scheme of Ar ion bounce, (**a**–**c**) process of bounce, (**d**–**f**) corresponding motion of Al atoms.

**Figure 3:**
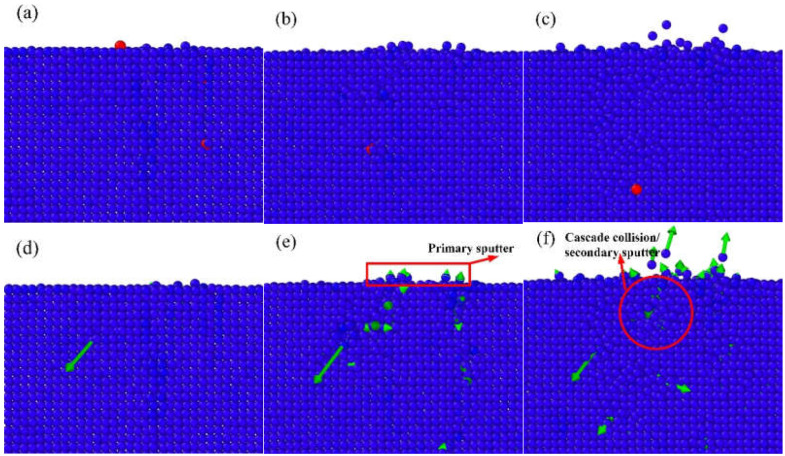
Scheme of Ar ion implantation, (**a**–**c**) process of implantation, (**d**–**f**) corresponding motion of Al atoms.

The IBS can be a complicated process. The Ar atoms bombard on the surface and perturb the Al atoms. The Al atoms receive enough energy and will be sputtered from the workpiece surface. During IBS, there will be two types of Ar ion behaviors: ion bounce and ion implantation, which are shown in Figure 2 and Figure 3, respectively. For ion bounce, the Ar atoms impact the aluminum surface and embed into the substrate. Few surface Al atoms are disturbed, as shown in Figure 2d. Then, the Ar ion collides with Al atom, with a rapid transfer of kinetic energy. The Ar ion bounces back and the recoil Al atom will continue the movement, as shown in Figure 2f. For ion bounce, few Al atoms are exposed by Ar atoms during the bombardment and there is a rapid exchange of kinetic energy during the impact.

Ion implantation will occur for most of the Ar atoms. As shown in Figure 3a,d, the implantation will cause the perturbation of the deeper Al atoms. With the motion of the Ar atoms, large numbers of Al atoms in the bombardment area gain kinetic energy. Some atoms are directly sputtered out after colliding with Ar atoms, as shown in Figure 3e, which is referred as primary sputter phenomenon. The Al atoms with kinetic energy will bump into other atoms in a cascade collision, which will cause secondary sputter phenomenon, as shown in Figure 3f.

### 3.2. Sputter Yield Analysis

**Figure 4:**
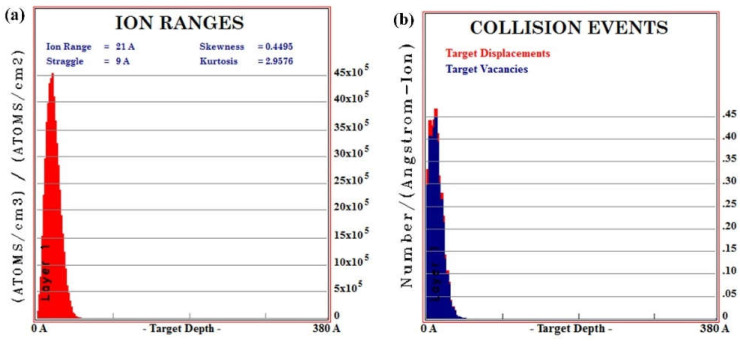
Monte Carlo simulation results of IBS process, (**a**) Ar atoms distribution, (**b**) damage distribution.

IBS is believed to possess the highest machining precision. The steady erosion rate of IBS can be expressed as follow:

(6) $$v_{0}=\Omega JY_{0}$$

where *J* is the ion current, Ω is the atomic volume, and *Y_0_* is the sputter yield. In order to achieve high precision material removal, it is necessary to acquire precise sputtered yield for corresponding materials. Usually, Monte Carlo methods are used to simulate sputter yields, which have a high accuracy for isotropic materials such as fused silica. However, the simulation setup of many Monte Carlo methods is relatively simple, which cause great deviation from experimental results. Commonly used Monte Carlo simulations for IBS are based on SRIM. Figure 4 shows the Monte Carlo simulation results of IBS of aluminum. With same simulation parameters, the Ar atoms mainly distribute in the depth of around 58 Å or less. The displacements and vacancies have the same distributions, which is similar to the Ar distribution. The sputter yield calculated by SRIM is 0.6707 Atoms/Ions. However, the IBS process of different crystal orientations cannot be revealed by SRIM, which slightly limits the application of the Monte Carlo simulation.

**Figure 5:**
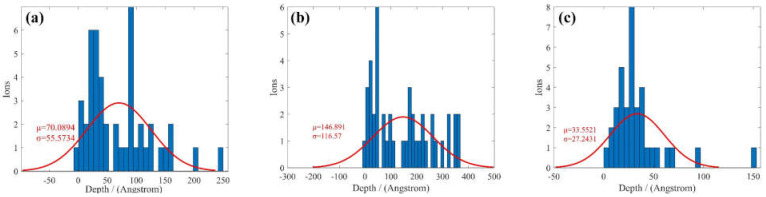
Ar atoms distribution and corresponding Gaussian distribution fitting of, (**a**) Al(001), (**b**) Al(110), (**c**) Al(111).

Based on MD simulation, the Ar atoms distribution and corresponding Gaussian distribution fitting in the z direction are presented in Figure 5. For Al(001), the average depth of bombardment is around 70 Å. As the depth increases, the number of Ar atoms decreases, which conforms to the actual processing conditions. Al(111) shares the same regularity with Al(001). However, according to the expectation and variance, the distribution of Ar atoms is more concentrated and the depth is shallower. For Al(110), Ar atoms are more evenly distributed relatively. The average depth of bombardment is 146 Å, which is the deepest among the three crystal orientations.

**Figure 6:**
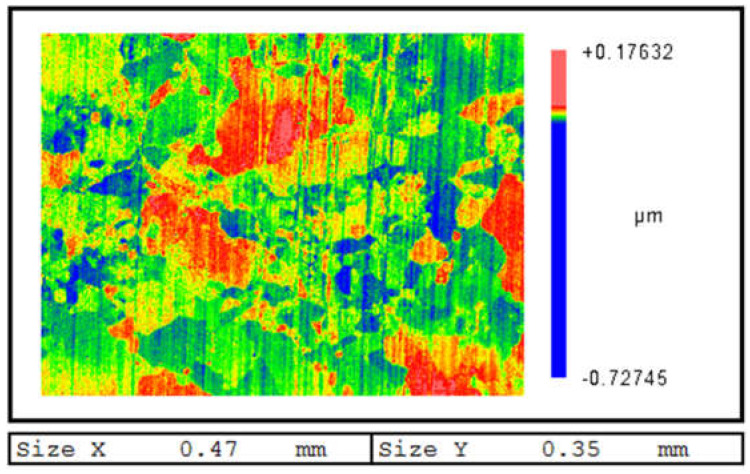
Surface morphology of aluminum with short time IBS.

The sputter yield can be calculated by the division of sputtered Al atoms and Ar ions. The sputter yields of Al(001), Al(110), and Al(111) are 1.24 atoms/ions, 0.84 atoms/ions, and 1.7 atoms/ions, respectively. The sputter yield of Al(111) is nearly twice that of the Al(110). The experimental result of IBS of polycrystalline aluminum is shown in Figure 6. The experiment is conducted on a *ϕ*100 mm planar aluminum surface. The surface is polished to roughness of 2 nmRa with no particular micro morphology. The processing parameters in Table 1 are used. The incident angle is 90°. When the processing time is short, the sputtering yield difference of different crystal surfaces will cause the obvious relief structure. The size of relief structure is similar to aluminum grains, which also verifies our analysis. Compared with Monte Carlo simulations, MD simulations are closer to the experiments, and are more comprehensive and accurate.

### 3.3. Surface Morphology and Roughness Evolution

**Figure 7:**
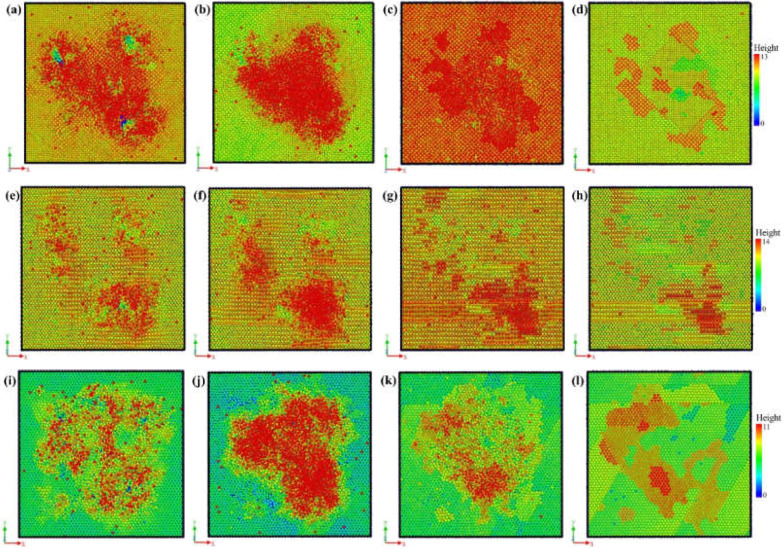
Surface morphology evolution of aluminum crystal surface of; Al(001) (**a**) 6 ps, (**b**) 9 ps, (**c**) 28 ps, (**d**) 100 ps; Al(110) (**e**) 6 ps, (**f**) 9 ps, (**g**) 28 ps, (**h**) 100 ps; Al(111) (**i**) 6 ps, (**j**) 9 ps, (**k**) 28 ps, (**l**) 100 ps.

**Figure 8:**
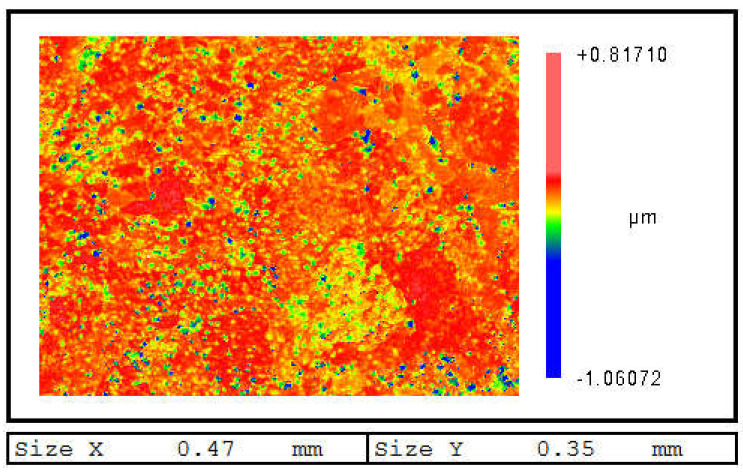
Surface morphology of aluminum with long time IBS.

Due to its highest machining precision, IBS is usually used in optics fabrication as the final process. Thus, the surface quality and morphology evolution are important concerns during IBS. Normally speaking, the surface quality can be preserved during IBS for many non-metallic materials such as fused silica and monocrystalline silicon. However, for aluminum, the surface quality deteriorates significantly during IBS in our previous experiments. Figure 7 shows the surface morphology evolution of different crystal surfaces. For Al(001), the evolution of morphology can be divided into four stages. Firstly, as shown in Figure 7a, the Ar atoms bombardment causes obvious pits on the surface. The surface atoms are disturbed and the fluctuation appears on the surface. The atoms outside the sputtering area are also affected by the bombardment and deviate from the original position. However, the bombardment and cascade collision process will end after few ps. Thus, the form of the morphology varies, as shown in Figure 7b. The atoms in the sputtering area are disturbed and an embossment is formed. Meanwhile, most of the atoms outside the sputtering area present a tendency to restore to the original state, which leads to the surface of an unbombarded area to flatten out. Then, with increasing relaxation time, the atoms diffusion dominates the evolution of surface morphology. The atoms outside the sputtering area are subject to secondary perturbation. The height of the embossment decreases with the diffusion and specific morphology emerging gradually. Finally, with long-time relaxation, the surface morphology with local gravel structures is formed and stabilized. As for the crystal surfaces of Al(110) and Al(111), the evolution of morphology can also be expressed by the same four stages. However, at the same stage, there is difference for different crystal surfaces. For Al(110), the atoms outside the sputtering area do not restore after disturbance, which highly affects the subsequent atoms diffusion. Thus, the surface morphology of Al(110) shows no obvious specific structure after stabilization. For Al(111), the atoms outside the sputtering area are barely affected by the bombardment and diffusion. By comparison, the stabilized morphology of Al(111) has the most obvious morphological features. With the processing time increasing, the crystal surface begins to coarse with the increasing bombardment atoms. The relief structure will gradually disappear and evolve into gravel structure, as shown in Figure 8. In Figure 8, the regionalization of morphology is also obvious. The number of gravels at a specific area are significantly larger than other areas. Based on above analysis, different crystal surfaces will have a large difference on the generation of gravels, which is in good agreement with experimental results.

**Figure 9:**
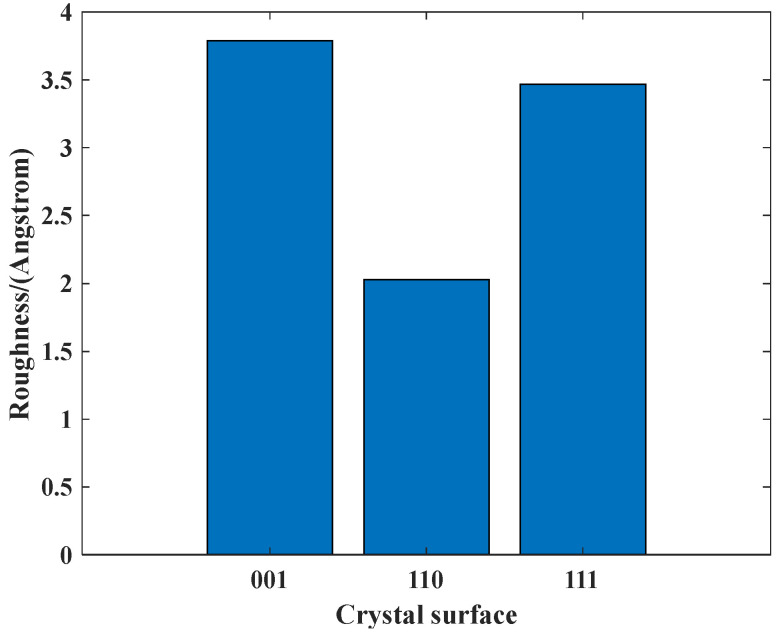
Roughness comparison of different crystal surfaces.

The surface roughness is an important characteristic for optical components. The surface contour arithmetic mean deviation *R_a_* is usually used to evaluate surface roughness, which can be expressed as follow:

(7) $$R_{a}=\frac{1}{l_{r}}\int_{0}^{l_{r}}{|y}_{i}|dx$$

where *y_i_* is the height of sampling point, and *l_r_* is the sampling length. After stabilization of system, the surface roughness is calculated for each crystal surface, as shown in Figure 9. The Al(110) has the lowest surface roughness of 2.02 Å. Al(111) and Al(001) have the surface roughness of 3.4 Å and 3.8 Å, respectively. For Al(110), the surface pits caused by bombardment is not obvious and the surface state is relatively uniform after surface disturbed. Thus, the final surface roughness of Al(110) is relatively better than the other two crystal surfaces. Comparing with Al(111), the atoms outside the sputtering area of Al(001) are easily disturbed and are harder to stabilize during relaxation, which will cause resistance to atom diffusion and surface roughness deterioration. Based on the above analysis, the surface-disturbed state and atom diffusion state will dominate the final surface roughness. The surface roughness of different crystal surfaces varies greatly. In actual IBS process of aluminum alloy, the surface is comprised of various crystal surfaces. Thus, the surface roughness of aluminum alloy is harder to maintain during the IBS process, which is quite consistent with the experimental results.

### 3.4. Subsurface Damage and Machining Stress Analysis

**Figure 10:**
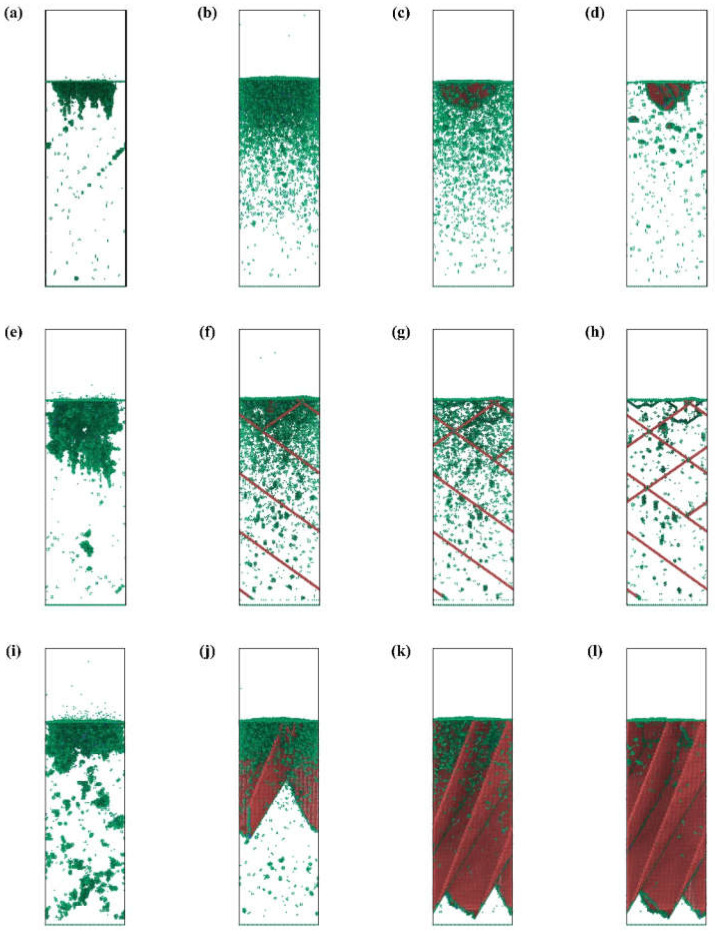
The evolution of subsurface damage, of Al(001) (**a**) 200 fs (**b**) 20,900 fs (**c**) 80,000 fs (**d**) 240,000 fs, of Al(110) (**e**) 200 fs (**f**) 20,900 fs (**g**) 80,000 fs (**h**) 240,000 fs, of Al(111) (**i**) 200 fs (**j**) 20,900 fs (**k**) 80,000 fs (**l**) 240,000 fs.

The subsurface damage and machining stress are important concerns in optics fabrication. The subsurface damage in the aluminum optics may cause the surface corrosion, which will severely destroy the surface quality. The subsurface damage evolution processes of the different crystal surfaces are displayed in Figure 10. The lattice defects are identified by the CNA method. The evolution of the subsurface damage can be roughly divided into four stages. Take Al(001) as an example, the bombarded Ar atoms firstly causes the disturbance of the subsurface. The bombardment will end quickly in less than 0.1 ps. The cascade collision of Al atoms will affect the deeper atoms, as shown in Figure 10b. Then, the stacking fault (SF) will generate with the system stabilizing. The disturbed Al atoms are reduced and the system can finally reach a stable state in Figure 10d. Comparing with Al(001), the disturbance layer of Al(110) and Al(111) are obviously deeper, which is consistent with the analysis in Section 3.3. The appearance of SFs in Al(110) and Al(111) is earlier than that of Al(001). After stabilization, the distribution of SFs in the Al(110) and the Al(111) are deeper than in the Al(001). The style of defects in the three crystal surface are consistent which is mainly SFs, but the distribution feature is quite different. For the Al(001), the SFs mainly concentrate in the bombardment area and have a shallow distribution. However, the SFs exist in a grid pattern and extend to a deeper location in the Al(110). For the Al(111), the SFs appear in the laminated structure and their amount is obviously highest.

**Figure 11:**
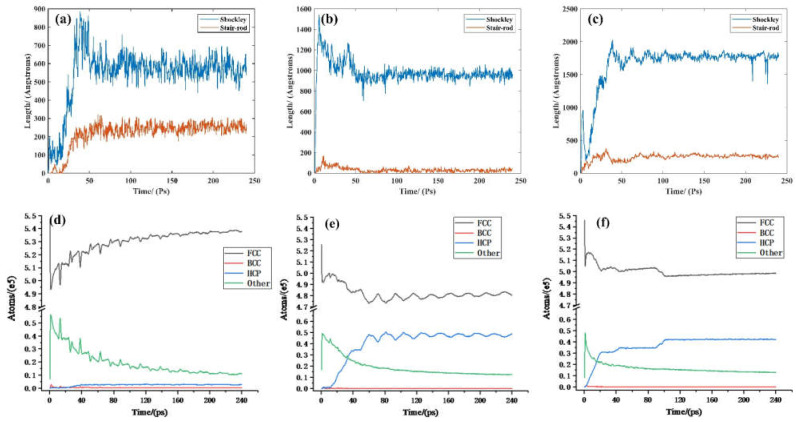
Dislocation statistics of, (**a**) Al(001), (**b**) Al(110), (**c**) Al(111); and atomic type statistics of, (**d**) Al(001), (**e**) Al(110), (**f**) Al(111).

Figure 11 gives the evolution of dislocation length and atom number in the Al(001), Al(110), and Al(111) during total IBS machining. The IBS mainly induces the Shockley dislocation and stair-rod dislocation generated in the Al(001), Al(110), and Al(111). It can be observed from Figure 11a–c that the dislocation length firstly increases and then tends to stabilize. Comparing the stable stages in the Figure 11a–c, the Shockley dislocations are more obvious in the Al(111) than in the Al(001) and Al(110), while the stair-rod dislocations are relatively more in the Al (111). The phenomenon demonstrates that, after IBS, the density of dislocations in the Al(111) is largest, thereby bringing about significant dislocation strengthening for the Al(111).

In addition, it can be seen from Figure 11d–f that the number of face-centered cubic (FCC) atoms rapidly increases with the abrupt decrease in the number of other atoms, which reveals that the FCC atoms in the workpiece are mainly turned into the other atoms due to the high speed intrusion of Ar atoms. The result is consistent with the phenomena shown in Figure 10a,e,i. With the time increasing, it is observed that the number of other atoms decreases gradually when that of hexagonal closepacked structure (HCP) atoms increases, indicating that the other atoms are further translated into the HCP atoms. As a result, the numbers of FCC, Other and HCP atoms obtain certain values. The number of HCP atoms is highest in the Al(111), which is consistent with the results in Figure 10.

The above phenomena indicate that the crystal orientation of a machined surface exert an apparent influence on the subsurface damage. For the three surfaces of Al(001), Al(110), and Al(111), the subsurface damage of Al(111) is severest, because the numerous SFs are generated and distributed in the deepest part of the workpiece.

**Figure 12:**
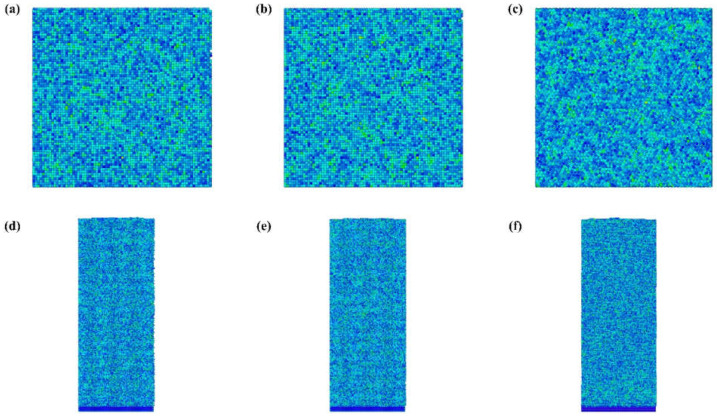
The stress distribution after relaxation, of Al(001) (**a**)surface (**d**) cross section, of Al(110) (**b**) surface (**e**) cross section, of Al(111) (**c**) surface (**f**) cross section.

Figure 12a–c show the stress distribution of the three crystal surface after IBS. There is no significant concentration of stress after IBS. In addition, Figure 12d–f display the cross section of stress distribution in the Al(001), Al(110), and Al(111) after IBS. Similarly, hardly any stress concentration is introduced to the workpiece by the IBS machining. The phenomenon demonstrates that the Ar ion beam bombardment causes no additional stress in the workpiece. In addition, it can also be used to release stress caused by other processing methods. Therefore, the IBS is believed to be stress-free machining in the optical fabrication field. The simulation results are quite consistent with the experimental results [17].

## 4. Conclusions

In this paper, the MD simulation of IBS process of aluminum with different crystal orientation is studied. Influences of different crystallographic orientations on IBS process and manufactured results are revealed.

Firstly, the ion beam sputtering mechanism is exposed. Two states of Ar atoms (implantation and bounce) are observed. The implantation of Ar atoms causes massive disturbance of the shallow Al atoms, which leads to primary sputtering effect. The shallow Al atoms will cause cascade collision and lead to secondary sputtering effect. The simulation results are consistent with the traditional sputtering theory, which verify the validity of the MD simulation.

Secondly, the sputter yield, morphology evolution, and surface roughness are revealed by simulation results. Three crystal surfaces show great variety. The sputter yield of Al(111) is nearly twice that of Al(110). When the processing time of IBS is short, the varied sputter yield of different crystal surfaces will cause the emergence of the relief structure. With increased bombard time, the gravel structure of single crystal surface will dominate the morphology evolution. The state of atom diffusion (during the bombardment and during the relaxation) determines the final morphology and roughness of specific crystal surfaces. With easier atom diffusion, Al(110) has the lowest roughness. However, with poor atom diffusion during the bombardment and large disturbance during relaxation, the roughness of Al(001) is nearly twice that of Al(110).

Finally, the subsurface damage and machining stress are analyzed. The main defects for different crystal surfaces are identical, which are stacking fault, Shockley dislocation, and stair-rod dislocation. However, the form and distribution show great difference. For Al(001), the defects generate on the bombardment area and have a shallow distribution. The defects are in a grid pattern and extend to a deeper location in Al(110). For Al(111), the defects have a laminated structure and have the highest amount. IBS is believed to be stress-free machining in the optical fabrication field. There are no significantly concentrations of stress after IBS for all three crystal surfaces, which is consistent with the experimental results.

The process of IBS and mechanism of morphology evolution of aluminum are revealed. The regularity and mechanism will lay a foundation for the application of IBS in aluminum optics manufacture fields.

## Figures and Tables

**Table 1:** Simulation parameters of ion beam sputtering.

Material	Aluminum
**Dimension**	15 × 15 × 38 nm^3^
**Number of atoms**	554,040
**Time step**	1 fs
**Initial temperature**	293 k
**Incident angle**	90°
**Ion energy (Ion velocity)**	500 eV (491 Å/fs)
**Lattice plane**	(110) (111) (001)
**Ion dose**	2.2 × 10^13^ ion/cm^2^
